# A huggable device can reduce the stress of calling an unfamiliar person on the phone for individuals with ASD

**DOI:** 10.1371/journal.pone.0254675

**Published:** 2021-07-23

**Authors:** Hidenobu Sumioka, Hirokazu Kumazaki, Taro Muramatsu, Yuichiro Yoshikawa, Hiroshi Ishiguro, Haruhiro Higashida, Teruko Yuhi, Masaru Mimura

**Affiliations:** 1 Hiroshi Ishiguro Laboratories, Advanced Telecommunications Research Institute International, Kyoto, Japan; 2 Department of Preventive Intervention for Psychiatric Disorders, National Center of Neurology and Psychiatry, National Institute of Mental Health, Tokyo, Japan; 3 Research Center for Child Mental Development, Kanazawa University, Kanazawa, Ishikawa, Japan; 4 Department of Neuropsychiatry, Keio University School of Medicine, Tokyo, Japan; 5 Department of Systems Innovation, Graduate School of Engineering Science, Osaka University, Osaka, Japan; Hamamatsu University School of Medicine, JAPAN

## Abstract

Individuals with autism spectrum disorders (ASD) are often not comfortable during mobile-phone conversations with unfamiliar people. “*Hugvie*” is a pillow with a human-like shape that has been designed to provide users with the tactile sensation of hugging another person during phone conversations to promote feelings of comfort and trust in the speaker toward their conversation partners. Our primary aim was to examine whether physical contact by hugging a *Hugvie* could reduce the stress of speaking with an unfamiliar person on the phone in individuals with ASD. We enrolled 24 individuals and requested them to carry out phone conversations either using only a mobile phone or using a mobile phone along with the *Hugvie*. All participants in both groups completed questionnaires designed to evaluate their self-confidence while talking on the phone, and also provided salivary cortisol samples four times each day. Our analysis revealed that the medium of communication was a significant factor, indicating that individuals with ASD who spoke with an unfamiliar person on the phone while hugging a *Hugvie* had stronger self-confidence and lower stress levels than those who did not use *Hugvie*. Hence, we recommend that huggable devices be used as adjunctive tools to support individuals with ASD during telephonic conversations with unfamiliar people.

## Introduction

Recent technological advancements in electronic devices and remote communication options, such as mobile phones, have dramatically impacted the way individuals communicate in daily life [1]. To participate in civil society today, it is necessary to use mobile phones to communicate with unknown people in a variety of situations. However, communication and language is a core challenge for individuals with autism spectrum disorders (ASD). Their barriers in communication lead to increased social withdrawal and avoidance [2]. They are often not comfortable talking to unfamiliar people on mobile phones, partly because their imagination level is low and their anxiety level is high.

Stress and anxiety make it difficult for people to exercise self-control and concentrate on their communication partners [3], and this problem is heightened for individuals with ASD due to their limited ability to exercise self-control. Psychological studies have suggested that tactile sensations increase feelings of comfort during communication [4–7] and that the physical presence of the conversation partner’s body significantly influences the speaker’s perception of the outside world [8, 9]. Interactions wherein people touch one another activate the tactile channel in the brain and reduces stress––a known effect of interpersonal touch [4]. Given these facts, there is increasing interest in reproducing the psychological effects associated with interpersonal touch by introducing tactile sensations within communication devices.

**Fig 1:**
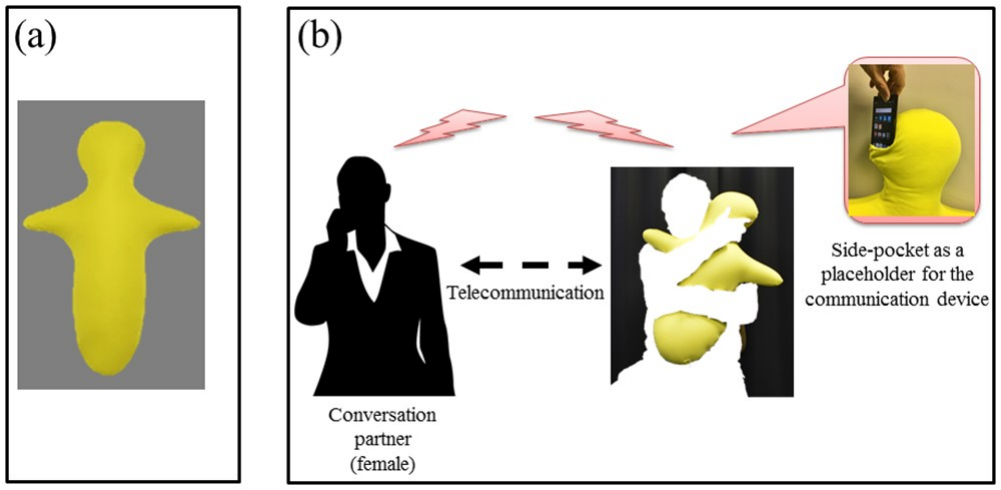
(a) *Hugvie*; (b) An individual telecommunicating with a remote person while hugging a *Hugvie*.

“*Hugvie*” (Fig 1) is a pillow shaped like a human that was designed to mimic the tactile sensation of hugging. It can be used by individuals while they are having a conversation on the phone and aims to promote positive feelings, such as comfort and trust, towards the person on the other end of the line [10–12]. Using a *Hugvie*, i.e., squeezing a human-like shape and hearing a voice near the ears, makes people feel like they are hugging the person on the phone. This allows users to feel connected with and experience the presence of their remote conversation partner [13].

Individuals with ASD tend to display abnormalities in processing tactile stimuli [14–19]. Because of this, it is not clear whether using a *Hugvie* is beneficial for individuals with ASD while speaking on the mobile phone with an unfamiliar person. Given the barriers that individuals with ASD experience while talking to unfamiliar people on mobile phones and the social dysfunction that characterizes ASD, we believe that *Hugvie* can effectively decrease stress in such interactions.

The primary aim of this study was to examine whether physical contact by hugging a *Hugvie* [9] can reduce stress in individuals with ASD as they speak on the phone with an unfamiliar person. Hugging a *Hugvie* while having a phone conversation reduces stress in the general population [9], and individuals with ASD generally judge textures to be more pleasant than controls do [20]. Given this, we hypothesized that the use of a *Hugvie* would also better prepare individuals with ASD for talking on the phone with an unfamiliar person.

In the present study, we employed self-reporting measures and measured salivary cortisol levels to determine whether the use of a *Hugvie* alters the self-confidence and stress metrics of individuals with ASD. The greater self-confidence in one’s ability to communicate with others, the greater is the individual’s communication performance [21–23]. Salivary cortisol is known as a reliable, noninvasive biomarker of stress that is used to evaluate feelings of stress in response to social situations [24–26]. A previous study [10] tested the utility of hugging *Hugvie* in the general population, wherein participants had 15-minute mobile-phone conversations with a remote partner that were either carried out while hugging a *Hugvie* or not. The researchers compared the levels of cortisol between participants who used a *Hugvie* and those who used only a phone during the conversation and showed that the concentration of cortisol decreased in the *Hugvie* participants. Cortisol levels are known to be related to self-confidence [27], and measuring the physiological arousal levels and assessing self-reports from individuals with ASD are imperative to obtain an accurate evaluation [23, 28, 29]. Therefore, we aimed to obtain an objective assessment of self-confidence in individuals with ASD by evaluating their self-reported and physiological (salivary cortisol) measures of arousal.

## Materials and methods

### Participants

For this study, we recruited young adults with ASD from Kanazawa University by placing printed posters at related institutions within the university. We received approval from the Ethics Committee of Kanazawa University and carried out our experiment as per the standards of the institutional and/or national research committee and the 1964 Declaration of Helsinki. All participants and their parents provided written informed consent and agreed to participate after receiving a complete explanation of the study. The inclusion criteria for the participants were as follows: (1) age of 15–24 years; (2) IQ ≥ 60; and (3) confirmation from experienced psychiatrists that the participants could understand and accurately complete the informed consent document, questionnaire, and experiment procedure. Experienced psychiatrists confirmed that all participants, including one individual whose IQ was lower than 70, were able to accurately give their written informed consent and understand the study method, questionnaire, and experiment procedure. After getting enrolled in the study, all the participants were evaluated by experienced psychiatrists and diagnosed as having ASD according to the criteria in the Diagnostic and Statistical Manual of Mental Disorders (DSM-5) [30] and the standardized criteria outlined in the Diagnostic Interview for Social and Communication Disorders (DISCO) [31]. The DISCO is an instrument of diagnosis with good psychometric properties that comprises items that assess early development and activities of daily life, allowing the interviewer to ascertain the level of functioning of ASD-affected individuals in several other areas apart from social functioning and communication [32]. We enrolled all participants who were determined to have childhood autism, atypical autism, or Asperger’s syndrome as per the DISCO. To exclude other psychiatric diagnoses, we administered the Mini-International Neuropsychiatric Interview (M.I.N.I.) [33], which revealed that none of the participants had other psychiatric disorders apart from ASD.

We used the Autism Spectrum Quotient-Japanese version (AQ-J) to evaluate ASD-specific behaviors and symptoms in each participant [34]. The AQ-J has been used in previous studies across different cultures [35]. The questionnaire is sensitive in defining the broader autism phenotype [36]. We additionally used the Wechsler Adult Intelligence Scale-Fourth Edition [37] to measure IQ in individuals with ASD.

We employed the Liebowitz Social Anxiety Scale (LSAS) [38] to measure the severity of social anxiety symptoms. There is a known correlation between the LSAS score and symptoms of social anxiety, wherein an LSAS score of 30 reflects minimal symptoms and is considered the optimal cut-off score to identify individuals with an anxiety disorder [39].

The ADHD Rating Scale (ADHD-RS) [40] comprises 18 items that assess inattentive and hyperactive-impulsive symptoms on a 4-point scale (0 = never, 1 = sometimes, 2 = often, 3 = very often) and assesses symptom severity over the past week. The total score is calculated by summing the individual scores of the 18 items.

The Adolescent/Adult Sensory Profile (AASP) [41] is a self-report questionnaire that measures sensory processing in individuals 11 years of age or older by examining four different “quadrants” of sensory processing (i.e., low registration, sensation-seeking, sensory sensitivity, and sensation avoidance). We administered the AASP to all participants and ensured that they completed it. Before commencing the experiment, we determined how often the participants exhibited certain behaviors related to sensory processing on a scale of 1 (almost never) to 5 (almost always).

### Procedure

We used a human-shaped pillow phone called *Hugvie* [10] (Nishikawa Co; length: 75 cm, weight: 600 g), a communication device designed to provide tactile stimulation to users. It is a soft pillow filled with polystyrene microbeads and covered with a mixed fiber comprising acrylic and rayon. The shape resembles a person opening their arms for a hug and the pillow thud simulates the experience of hugging while having a conversation. The telecommunication is facilitated by a mobile phone placed inside a pocket near the pillow’s head. Because the phone is placed in the pocket, people can talk without holding the phone and can use their hands to engage with the *Hugvie*, thereby promoting the sensation that they are hugging their conversation partner. Because a *Hugvie* has no actuators inside it, we were able to investigate the effect of its passive contact with the user.

This was a crossover study, wherein the participants had a phone conversation with a partner they had never met in person while either hugging a *Hugvie* equipped with a mobile phone or using only a mobile phone. The unfamiliar phone partner was a 34-year-old female secretary who was employed by an institution to which some authors belong. She had no specialized professional skills to communicate with individuals with ASD. She was the phone partner in every conversation with participants from both groups. During each session, she initiated the conversation by asking questions based on a specific protocol. The scripts varied slightly across sessions to promote engagement but followed the same basic structure (examples of scripts in S1 File). The phone partner did not know whether the participant was using a *Hugvie* or only a mobile phone.

**Fig 2:**
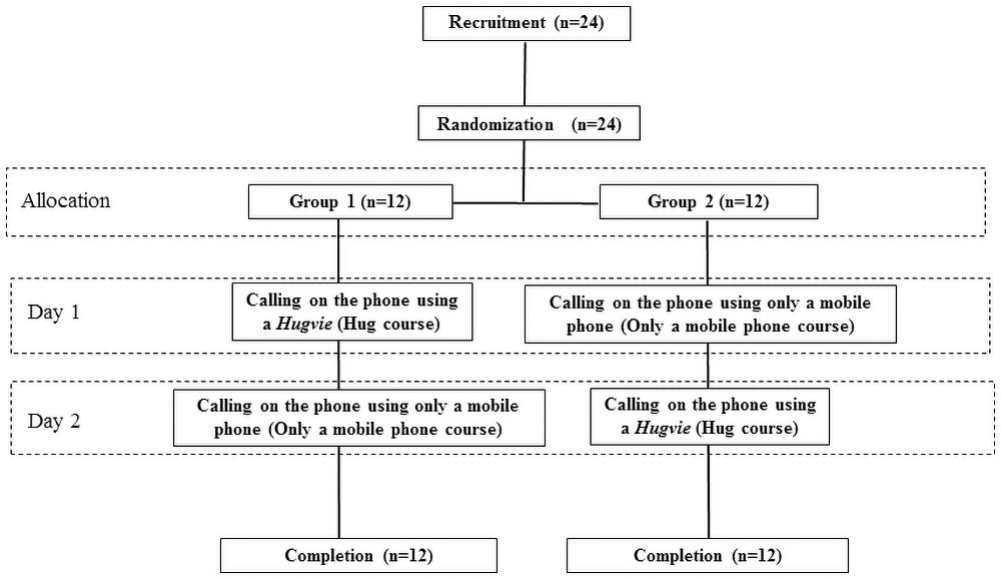
Participant recruitment flowchart. Initially, the participants were randomly assigned to two groups: Group 1 talked on the phone using a *Hugvie* on Day 1 and used only a mobile phone on Day 2, while Group 2 talked on the phone using only a mobile phone on Day 1 and talked on the phone using a *Hugvie* on Day 2. All participants completed the trial procedures.

The trial procedures were conducted for two consecutive days (i.e., Day 1 and Day 2). To reduce sequence effects, we counterbalanced the trial conditions between the two groups (Fig 2). The participants in the first group (Group 1; n = 13) talked on the phone while hugging a *Hugvie* on Day 1 and used the mobile phone only on Day 2, while the Group 2 participants (n = 11) followed the opposite conditions. All participants talked with the same partner on both days, and the partner did not allow for any awkward silence. The average duration of each phone conversation was approximately 10 minutes. To avoid the influence of diurnal rhythms on hormones, we ensured that each participant held their two phone conversations at the same time on Days 1 and 2. No participant faced any technical difficulties while using the *Hugvie* or the mobile phone.

After each phone conversation, either using only a mobile phone or the *Hugvie*, all participants completed questionnaires designed to assess their self-confidence during both conditions. The questionnaires were scored using a Likert rating scale ranging from 0 (not at all comfortable) to 6 (very comfortable). In addition, after completing both trial conditions, all participants answered a yes/no question: “Was it better for you to have a mobile phone conversation while hugging *Hugvie* compared to only a mobile phone?”

**Fig 3:**
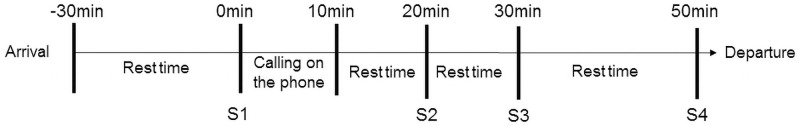
Timeline of events on Days 1 and 2. Both groups of participants (*Hugvie* users and only mobile phone users) were requested to relax for 30 minutes before and 40 minutes after talking on the phone. Salivary cortisol measures were collected just before the start of the phone conversation as a baseline measurement (S1), 20 minutes after the start of the conversation (S2), 20 minutes after the end of the conversation (S3), and 40 minutes after the end of the conversation.

To evaluate the participants’ physiological responses, we collected saliva samples to measure their cortisol levels on Days 1 and 2. Notably, there was an approximately 20-minute time lag between the occurrence of an event and the detection of event-related changes in salivary cortisol levels [42]. During the interaction, we collected four salivary cortisol samples from each subject after allowing for a time gap of 20 minutes. The collection intervals were S1 (just before the start of the phone conversation), S2 (20 minutes after the start of the phone conversation), S3 (20 minutes after the end of the phone conversation), and S4 (40 minutes after the end of the phone conversation). The S1 measurements were baseline values representing the circulating cortisol levels at resting time, while the S2, S3, and S4 measurements represented circulating cortisol levels at the start, end, and sometime after the experience, respectively (Fig 3). We collected the saliva samples from the participants at the same time each day to avoid potential diurnal variation and requested the participants to refrain from eating 1 hour before the phone conversation. Immediately before the phone conversation, we assigned each participant to a private room where a research assistant collected their saliva sample. We ensured that the participants were given time to relax for 30 minutes before and 40 minutes after the phone conversation in this room. The participants left the room only after they provided the last saliva sample for the S4 measurement.

The passive saliva samples (0.5–2.0 ml) were collected and immediately placed into sterile plastic tubes (15 ml), which were frozen on dry ice and stored at -80°C until use. After thawing at room temperature, the samples were centrifuged at 1500 × g for 10 minutes at 4°C to remove large precipitants. The salivary cortisol level was measured in duplicates using a cortisol enzyme immunoassay kit (Salimetrics, State College, PA, USA). The sample (25 μl) treatment was based on the manufacturer’s instructions. We used a microplate reader (Model 680, Bio-Rad, Richmond, CA, USA) to determine the optical density and standards of the samples at 450 nm. We also calculated the concentrations using MATLAB 7 based on the relevant standard curve [43].

### Data analysis

We performed the statistical analyses using SPSS version 24.0 (IBM, Armonk, NY, USA). Group differences in demographic data (age, full-scale IQ, AQ-J score, LSAS score, ADHD-RS score, and AASP subscale scores) were tested using the t-test. The difference in the proportions of males and females was analyzed using the χ2-test. We analyzed self-confidence ratings and the changes in salivary cortisol levels relative to those at baseline (S2/S1, S3/S1, and S4/S1) following the basic statistical procedure for crossover trials proposed previously [44].

We first tested our assumption that carryover effects (i.e., the effects carried over from one condition to another condition) were negligible using a t-test for within-subject sums of the values of the data for both groups on both days (data values from Day 1 + data values from Day 2). When the validity of the assumption was confirmed, we ran a t-test for within-subject differences between the results for both groups on Day 1 and Day 2 (data values from Day 1 –data values from Day 2) to evaluate the differences in treatment effects between the two communication media. We used t-tests for both the carryover and treatment effect analyses. The significance level for these analyses was 0.05.

## Results

### Demographic data

In total, 24 individuals with ASD participated in this study. The IQ of one participant was lower than 70, 17 participants had unusually high scores in the AQ-J, and 24 participants had social anxiety based on their LSAS scores. No participants had unusually high scores in the ADHD-RS. Six participants had unusually high scores in the Low registration, 2 had sensation-seeking tendencies, 5 had sensory sensitivity, and 6 had sensory avoiding (Table 1). All the participants completed the experimental procedure and the questionnaires. In response to the question, “Was it better for you to have a mobile phone conversation while hugging *Hugvie* compared to only a mobile phone”, 21 participants (87.5%) indicated “yes”, i.e., using *Hugvie* was better than using only a mobile phone. Three participants answered, “I liked neither of them.”

**Table 1:** Descriptive statistics of participants in group 1 and group 2 (n = 24).

Characteristics	Group 1 (n = 13)	Group 2 (n = 11)	Statistics
	M (SD)	M (SD)	*p*
Age in years	20.3 (3.4)	20.2 (2.4)	0.92
Gender (Male: Female)	11:2	10:1	0.64
Full-scale IQ	88.4 (14.4)	86.9 (14.4)	0.81
AQ-J	31.2 (4.3)	34.1 (3.2)	0.08
LSAS	47.4 (8.7)	43.0 (10.0)	0.27
ADHD-RS	8.2 (3.5)	9.7 (5.4)	0.42
AASP			
Low Registration	36.4 (7.4)	33.2 (9.3)	0.36
Sensation Seeking	37.2 (10.8)	33.0 (5.7)	0.26
Sensory Sensitivity	37.7 (9.6)	32.1 (11.5)	0.21
Sensation Avoiding	37.5 (10.4)	37.0 (12.4)	0.91

*Note*: M = mean, SD = standard deviation.

AQ-J = Autism Spectrum Quotient-Japanese version. In the AQ-J, higher scores reflect a greater number of ASD-specific behaviors.

LSAS = Liebowitz Social Anxiety Scale.

AASP = Adolescent/Adult Sensory Profile.

### Main result

**Fig 4:**
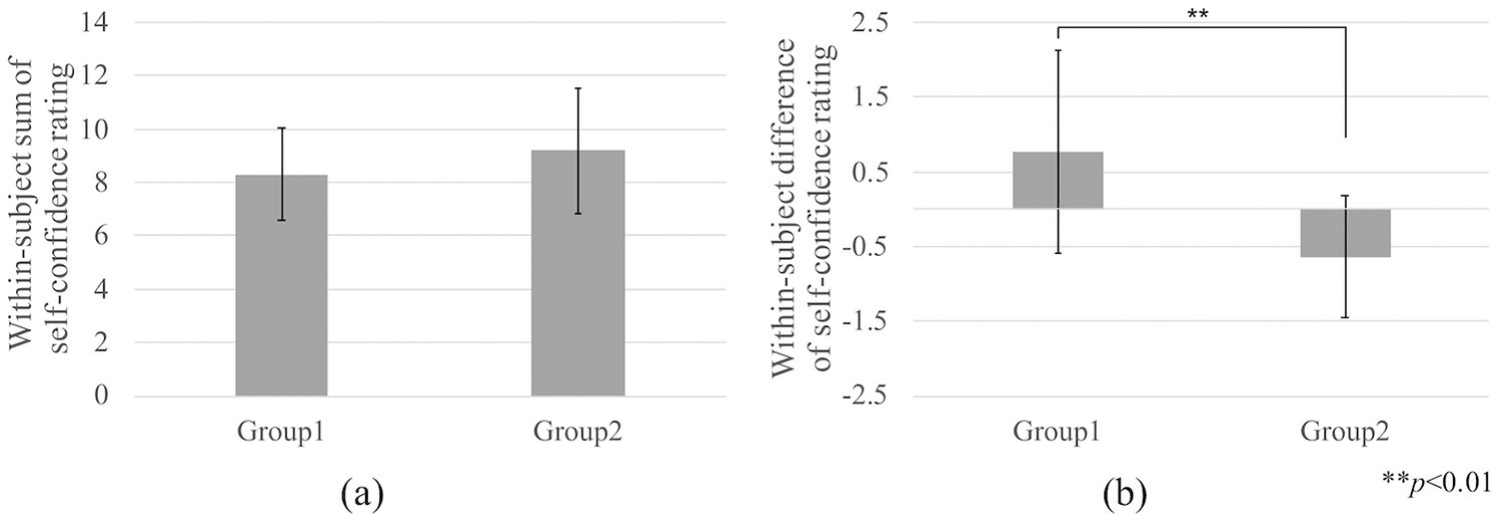
(a) Within-subject sum of the self-confidence ratings on Day 1 and Day 2; (b) Within-subject difference in the self-confidence ratings between Day 1 and Day 2.

Fig 4 shows the within-subject sum of self-confidence ratings from Day 1 and Day 2 for each group. Our analysis showed no evidence of relevant carryover effects (t (18.2) = -1.02, *p* = 0.32, *r* = 0.23). Therefore, we tested the treatment effect using the within-subject difference in self-confidence ratings between Day 1 and Day 2 for each group (Fig 4). There was a significant difference between groups (t (19.9) = 3.12, *p* = 0.01, *r* = 0.57), which indicated a definite improvement in the self-confidence rating in the participants that used the *Hugvie* as compared to those who used only the mobile phone.

**Fig 5:**
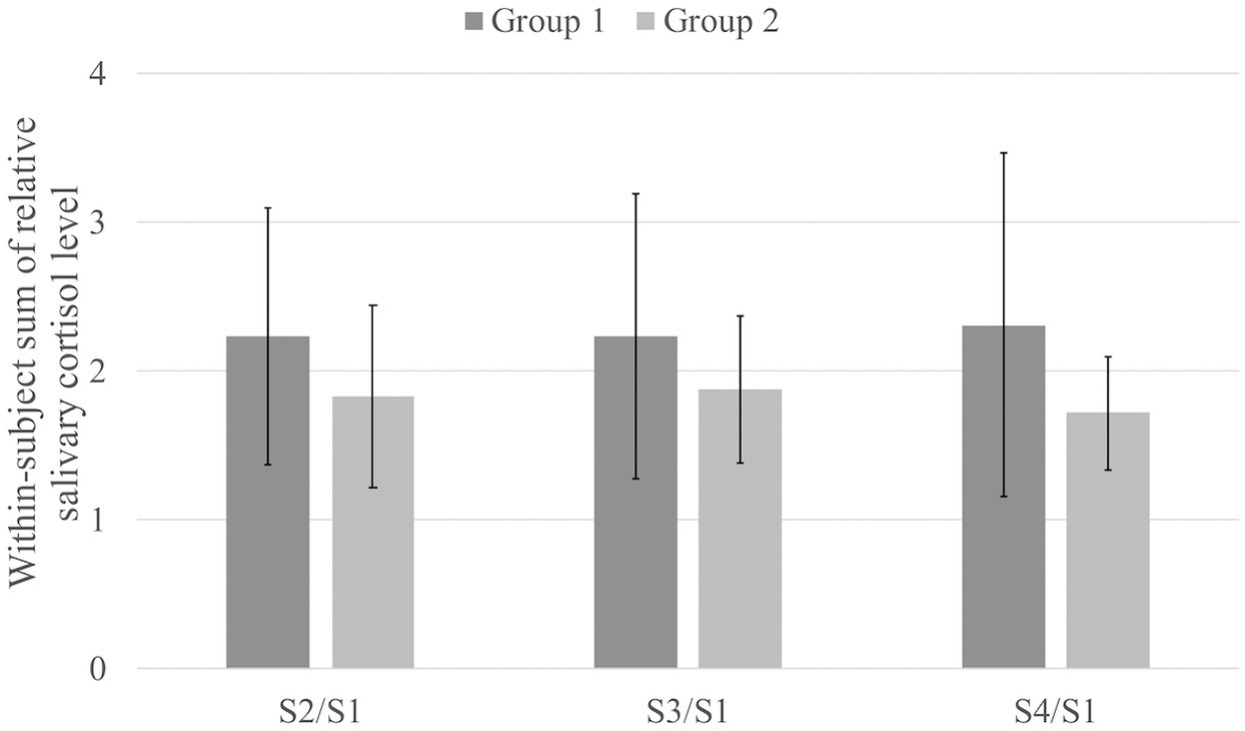
The within-subject sum of the changes in salivary cortisol levels on Day 1 and Day 2.

Fig 5 shows the within-subject sum of the changes in salivary cortisol levels on Day 1 and Day 2 for each sampling point (i.e., S2, S3, and S4) in each group. Our analysis of the carryover effects revealed no evidence of relevant carryover effects on changes in salivary cortisol levels for all the sampling points (S2/S1: t (21.4) = 1.34, *p* = 0.19, *r* = 0.28; S3/S1: t (18.4) = 1.17, *p* = 0.26, *r* = 0.26; and S4/S1: t (14.9) = 1.74, *p* = 0.10, *r* = 0.41). We also tested the treatment effect for all sampling points using the differences in changes in salivary cortisol levels between Day 1 and Day 2 for both groups.

**Fig 6:**
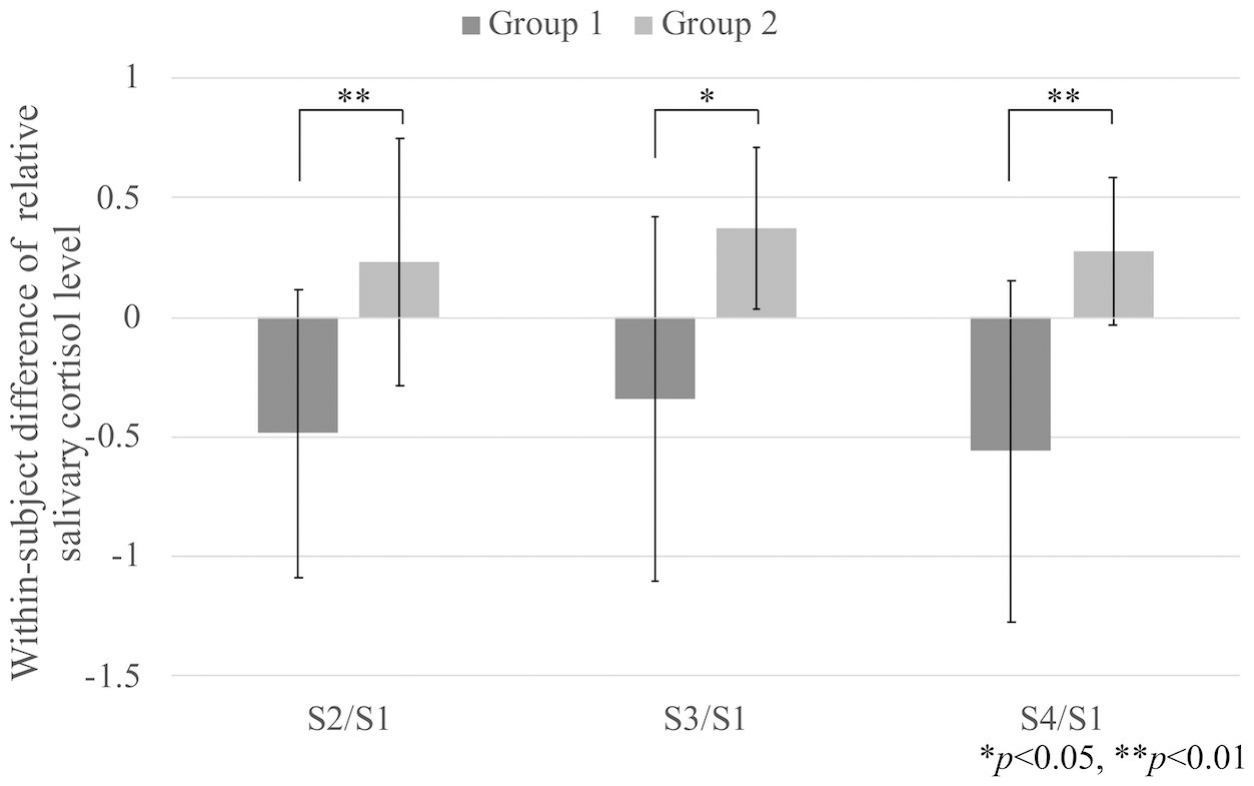
The within-subject difference in the changes in salivary cortisol levels between Day 1 and Day 2. The participants in Group 1 called an unfamiliar person on the phone while hugging a *Hugvie* on Day 1, followed by a phone call without a *Hugvie* (only using a mobile phone) on Day 2. The participants in Group 2 followed the opposite conditions on both days.

There were significant differences in the changes in salivary cortisol levels between participants using a *Hugvie* and those using only a phone for all sampling points (S2/S1: t (22.0) = -3.14, *p* = 0.05, *r* = 0.56; S3/S1: t (17.1) = -3.04, *p* = 0.01, *r* = 0.59; and S4/S1: t (16.9) = -3.82, *p* = 0.01, *r* = 0.68). Fig 6 shows the within-subject differences in the changes in salivary cortisol levels between Day 1 and Day 2 for each sampling point in each group. Details regarding participants’ confidence rating after making a phone call and the changes in cortisol levels are described in Table 2.

**Table 2:** Means and standard errors of the mean of the confidence rating scale after the phone call and changes in cortisol levels in *Hugvie* users and only-mobile-phone users in Group 1 and Group 2.

	Group	Day	
		Day 1	Day 2
		(M, SEM)	(M, SEM)
**Self-confidence**			
	Group 1	4.54(1.05)	3.77 (1.17)
	Group 2	4.27 (0.38)	4.91 (0.37)
**Change in cortisol level S2/S1**			
	Group 1	0.87 (0.10)	1.36 (0.18)
	Group 2	1.03 (0.14)	0.80 (0.10)
S3/S1			
	Group 1	0.94 (0.09)	1.29 (0.22)
	Group 2	1.12 (0.10)	0.75 (0.08)
S4/S1			
	Group 1	0.87 (0.11)	1.43 (0.24)
	Group 2	1.00 (0.08)	0.72 (0.06)

M: mean; SEM: standard error of the mean.

## Discussion

In this study, we assessed the benefits of using a huggable pillow while simultaneously talking on the phone with an unfamiliar person in young adults with ASD. Our analysis showed that the pillow communication device, *Hugvie*, significantly reduced feelings of stress before and after the phone conversations, evidenced by decreased levels of cortisol in the participants’ saliva at all sampling times. There was also a significant improvement in the self-confidence of those who used the *Hugvie* as compared to those who used only the mobile phone. These results indicate that using a *Hugvie* during the conversation decreased stress and increased self-confidence. Thus, the study demonstrated that using a *Hugvie* while conversing with unfamiliar people could be beneficial, thereby emphasizing the importance of providing tactile stimulation to young adults with ASD in communication situations.

Young adults with ASD can report their psychiatric symptoms, including anxiety [45]. Self-reports of their mood dysregulation are thought to be more accurate than accounts given by their caregivers [46]. To corroborate this, the self-report questionnaire we used in this study showed improved self-confidence in participants who talked on the phone using a *Hugvie* compared to those who used only a mobile phone. This proves that the self-report method is highly reliable.

We also attempted to objectively ascertain the levels of self-confidence in our young adult participants by measuring their salivary cortisol levels. The level of physiological arousal in response to social interaction is significantly higher in individuals with ASD than in the general population [25, 26, 46, 47] and the time lag between the occurrence of an event and the detection of a related change in salivary cortisol is approximately 20 minutes [42]. In this study, we found significant differences in the cortisol response between those who used a *Hugvie* and those who used only a mobile phone at all saliva-sampling times and measurements (S2/S1, S3/S1, and S4/S1). These results indicate that hugging a *Hugvie* reduces stress after talking on the phone with an unfamiliar person.

In the literature, tactile input is classified as active touch or passive touch [48, 49]. Passive touch involves passively applying a tactile stimulus on the skin without voluntary movement, whereas active touch uses voluntary movement to seek the tactile stimulus, thereby tactile and proprioceptive information generated by the movement are inputted. When an individual is touched passively, they tend to be hypersensitive and cannot focus on the properties of the stimulus [50]. *Hugvie* is an instrument that urges active touch. By actively touching *Hugvie*, ASD-affected individuals are able to focus on its properties and become comfortable. Given this, it is possible that actively touching the *Hugvie* (and not passive touch) contributes to the results of this study.

Even in the general population, calling someone while hugging a *Hugvie* as compared to only using a phone leads to reduced cortisol levels; however, this difference is not significant [10]. In contrast, our results revealed subjective changes in our participants who used the *Hugvie* as compared to those who did not. The perception of touch differs between individuals with ASD and those with typical development [51–55]; presumably, individuals with ASD generally judge textures to be more pleasant [20]. This explains why our ASD-affected participants showed a more significant response to hugging the device.

There is also a high variability in the likes and dislikes of some textures among individuals with ASD as compared to those with typical development [56]. This finding is understandable considering that in general, individuals with ASD have strong likes and dislikes [57]. In this study, we presumed that the texture of *Hugvie* would suit our participants’ preferences. We believe that the *Hugvie* can be further improved and developed by striving for optimum texture.

It can be argued that the cortisol reduction observed in this study was due to the novelty of the *Hugvie*. However, we believe that the novelty effect was not dominant. A previous study reported that long-term use of *Hugvie* maintained improved listening performances in school children with special needs over three months. Furthermore, the children indicated a preference for using the *Hugvie* even after the experiment [58]. Although the demographic characteristics of our participants were different from those of the schoolchildren in terms of target age and symptoms, they too indicated a positive impression of the *Hugvie*. Therefore, it is likely that the effect of the *Hugvie* is persistent over time and is not a one-time novelty. To confirm the persistence of the effect, we recommend that the long-term impacts of the *Hugvie* on individuals with ASD be investigated in the future.

We would like to acknowledge several limitations of our study. First, our sample size is relatively small. Larger sample sizes are necessary to provide more meaningful salivary cortisol and self-report questionnaire data. In addition, most of our participants were male. Although there is a strong male bias documented among individuals with ASD, there are reported sex differences in sensory symptoms in individuals with ASD [59, 60]; therefore, future research should include more female participants. Second, the duration of our intervention (talking on the phone) was comparatively short; however, we judged that 10 minutes per session would be appropriate to meet the specific needs of individuals with ASD. Besides, all our participants were able to complete the trial. Third, we only included individuals with ASD. To elucidate the relationship between hugging a *Hugvie*, stress reduction, and increase in self-confidence more clearly, it is important to study individuals without ASD and compare their data with those of individuals with ASD. Fourth, the target age of the ADHD-RS questionnaire is 5–18 years. We judged that our participants could not assess their inattentive and hyperactive-impulsive symptoms by themselves because their metacognition was low. However, the parents of all our participants were familiar with these symptoms. Thus, although we recognize that ADHD-RS is not standardized in individuals over 19 years old, we had no choice but to use ADHD-RS questionnaires that were rated by our participants’ parents to assess their inattentive and hyperactive-impulsive symptoms timely and efficiently. Finally, because our participants used the *Hugvie* only once in this study, it is unclear whether they would have responded similarly over multiple sessions. While we did not test habituation effects in this study, it is one of the first systematic investigations on the effects of huggable devices on self-confidence and stress in individuals with ASD. Future investigations on the effects of using *Hugvie* multiple times may offer a more comprehensive understanding of the effects of habituation to the device over time.

## Conclusions

As hypothesized, we found positive evidence to suggest that individuals with ASD who talked on the phone with an unfamiliar person while hugging a *Hugvie* had stronger self-confidence and less stress as compared to those who used only the mobile phone. It is difficult for individuals with ASD to talk on the phone with an unfamiliar person, and having a source of tactile stimulation reduces this uncomfortable feeling. Given the results of this study, we recommend that huggable devices be used as adjunctive tools to support individuals with ASD when they talk on mobile phones to unfamiliar people. Our findings contribute meaningfully to the literature dedicated to designing interventions to overcome communication difficulties in individuals with ASD.

## Supporting information

S1 File Examples of conversation scripts. (DOCX)
