# Supplementary material for: A huggable device can reduce the stress of calling an unfamiliar person on the phone for individuals with ASD
Source: PLoS One. 2021 Jul 23;16(7):e0254675. doi: 10.1371/journal.pone.0254675 (PMC8301655; doi:10.1371/journal.pone.0254675)
Supplement: S1 File — (DOCX) [file pone.0254675.s001.docx]

**Sample scripts**

We used two different scripts in the experiment. Each script was used on different days for each subject. Therefore, the subject was not asked the same question twice by the conversation partner. The order of the scripts was counterbalanced by subjects. Some contents were adapted according to the order in which the scripts were presented; for example, the partner did not ask the subject’s name on the second day. The conversation partner also encouraged the subject to talk by asking “Could you tell me in more detail?”

**<Script A>**

1. Hello, this is Sato. Nice to meet you.
2. (In case you do not know the subject’s name) Can I ask your name?
3. I live in Kyoto. It is very cold/cold/warm/very warm here today. How about over there?
4. Which do you like better, cold days or hot days?
5. So please tell me about yourself.
6. What is your favorite food?
   - Why do you like it?
   - What food do you dislike?
   - Why do you dislike it?
7. What do you usually do at school?
   - What are you doing in special class?
   - Why did you select this class?
   - What other classes could you select?
8. Did you go on a school trip last year? What did you do during the school trip?
   - What was your aim on the school trip?
   - What were some good or bad memories from the school trip?
9. What do you want to do after graduation?
   - Why do you want to do it?
   - Do you have an actual plan for doing it?
10. What do you value?
    - How long has this been valuable to you?
11. Do you keep anything secret from your teachers?
    - Why do you keep it secret?

*The conversation partner asked the following questions of subjects when she had finished asking the questions listed above.*

1. Do you play video games?
   - What is your favorite game?
2. Do you like singing karaoke?
   - What do you sing?
3. I found a class named “FUREAI IGO” on the website of your school. Is it different from the game of Go (IGO)? Do you like it?
4. Could you tell me about Mr. XXX (teacher’s name)?
5. What do you do on your days off?
   - Why do you do it?

**<Script B>**

1. Hello, this is Sato. Nice to meet you.
2. (In case you do not know the subject’s name) Can I ask your name?
3. I live in Kyoto. Your place is far from Kyoto. It takes about four hours by train five hours by car to get there.
4. By the way, do you like riding in a vehicle?
5. So please tell me about yourself.
6. What is your favorite color?
   1. Why do you like it?
   2. What color do you dislike?
   3. Why do you dislike it?
7. What do you usually do at school?
   1. What do you do on your lunch break?
   2. Why do you do it?
   3. What do other classmates do on their lunch break?
8. Does your school have art shows?
   1. What did you show in the art shows?
   2. What did your classmates do for the event?
   3. What were the best memories from the event?
9. What do you want to do or become after graduation?
   1. Why do you want to do or become that?
   2. Do you have an actual plan to do or become that?
10. Do you have anything that you are particular about?
    1. How long have you been particular about it?
11. Do you keep anything secret from your family?
    1. Why do you keep it secret?

*The conversation partner asked the following questions of the subjects when she finished asking questions listed above.*

1. Do you have a favorite sport?
   1. What is it?
2. Do you like using a smart phone?
   1. What do you watch on it?
3. When time does your school day end? What do you do in the evening?
4. Could you tell me about other special events at your school?
5. Could you tell me about Mr. XXX (teacher’s name)?
